# A Regulatory Transcriptional Loop Controls Proliferation and Differentiation in *Drosophila* Neural Stem Cells

**DOI:** 10.1371/journal.pone.0097034

**Published:** 2014-05-07

**Authors:** Tetsuo Yasugi, Anja Fischer, Yanrui Jiang, Heinrich Reichert, Juergen A. Knoblich

**Affiliations:** 1 Institute of Molecular Biotechnology of the Austrian Academy of Sciences, Vienna, Austria; 2 Biozentrum, University of Basel, Basel, Switzerland; Academia Sinica, Taiwan

## Abstract

Neurogenesis is initiated by a set of basic Helix-Loop-Helix (bHLH) transcription factors that specify neural progenitors and allow them to generate neurons in multiple rounds of asymmetric cell division. The *Drosophila* Daughterless (Da) protein and its mammalian counterparts (E12/E47) act as heterodimerization factors for proneural genes and are therefore critically required for neurogenesis. Here, we demonstrate that Da can also be an inhibitor of the neural progenitor fate whose absence leads to stem cell overproliferation and tumor formation. We explain this paradox by demonstrating that Da induces the differentiation factor Prospero (Pros) whose asymmetric segregation is essential for differentiation in one of the two daughter cells. Da co-operates with the bHLH transcription factor Asense, whereas the other proneural genes are dispensible. After mitosis, Pros terminates Asense expression in one of the two daughter cells. In *da* mutants, *pros* is not expressed, leading to the formation of lethal transplantable brain tumors. Our results define a transcriptional feedback loop that regulates the balance between self-renewal and differentiation in *Drosophila* optic lobe neuroblasts. They indicate that initiation of a neural differentiation program in stem cells is essential to prevent tumorigenesis.

## Introduction

**Figure 1:**
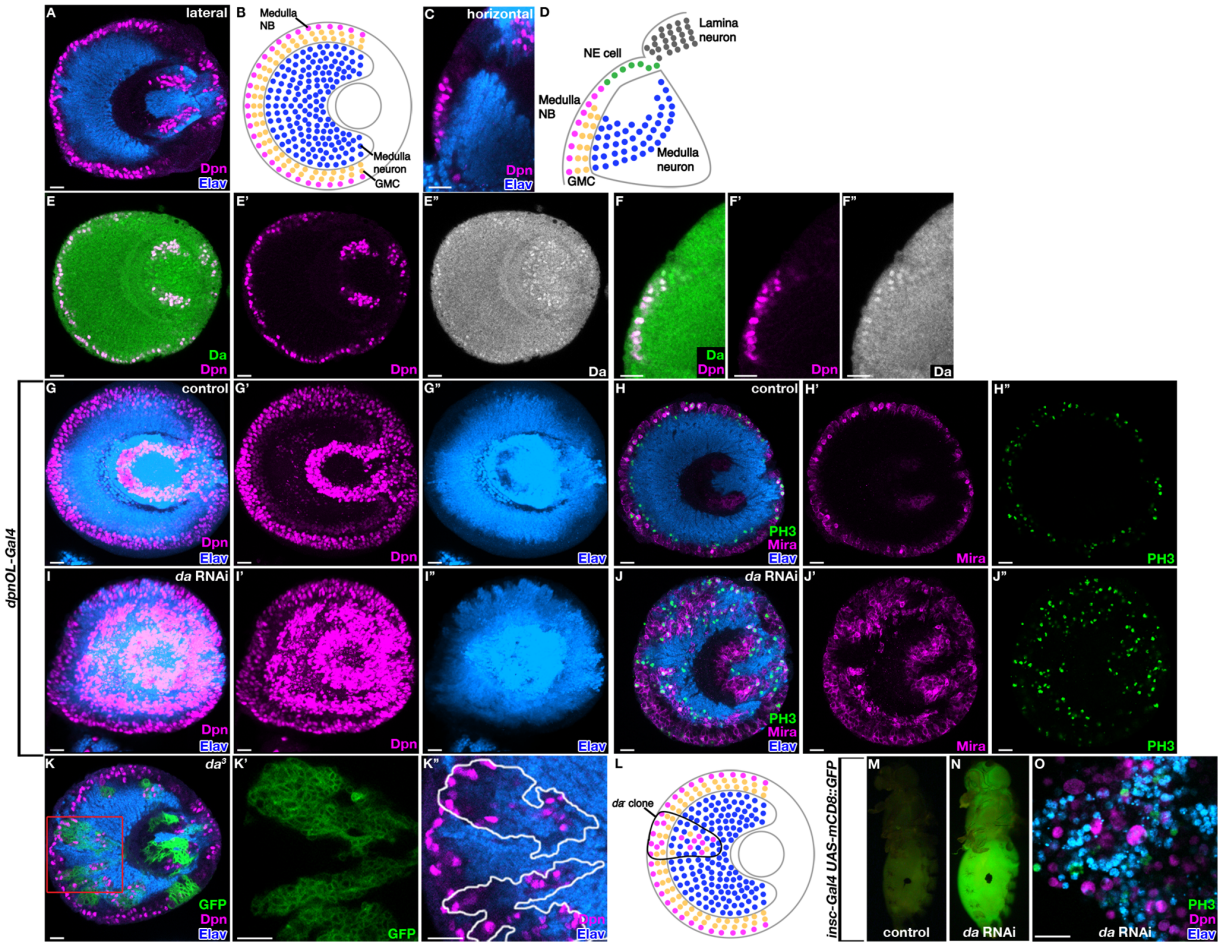
*da* is required for the cell fate determination in the optic lobe. (A) Lateral section of the optic lobe. (B) Schematic of the lateral section. Medulla NBs (magenta), GMCs (yellow), medulla neurons (blue) are indicated. (C) Horizontal section of the optic lobe. (D) Schematic of the horizontal section. Medulla NBs (magenta), GMCs (yellow), medulla neurons (blue), NE cells (green), and lamina neurons (grey) are indicated. (E and F) Expression of Da in the optic lobe. Lateral section (E) and horizontal section (F) are shown. (G–J’’) Third instar larval brains for control (G and H) and *da* RNAi (I and J). *dpnOL-Gal4* was used as a *Gal4* driver. (K) *da^3^* mutant clones. (K’ and K’’) Enlarged view of the boxed region in (K). Clones are marked by GFP (K, K’) or outlined (K’’). (L) Schematic of the phenotype of *da^3^* mutant clones. Used colors are indicated in (B). (M–O) Transplantation experiments for control (M) and *da* RNAi (N and O) samples. *UAS-dicer2; insc-Gal4 UAS-mCD8::GFP* flies were used as a *Gal4* driver. (O) Tissue staining from a tumor sample by *da* RNAi transplantation. Weak auto-fluorescence from GFP can be seen in some of the cells with Dpn staining. Markers are as indicated in all figures. Scale bars, 20 μm.

Stem cells are defined by their ability to self-renew and produce differentiating daughter cells. These two features must be tightly controlled since misregulation can lead to stem cell loss and tissue degeneration or overproduction of stem cells and tumor formation. *Drosophila* neural stem cells called neuroblasts (NBs) are a well studied model system for investigating molecular and cellular mechanisms of stem cell maintenance and tumorigenesis as their mode of cell division and cell fate determination are well defined [1], [2]. In the larval brain several types of NBs are defined by their locations and ways of cell division [3]. NBs in the central brain delaminate from the ventral neuroectoderm during embryogenesis and are subdivided into type I and type II NBs [2]. Type I NBs divide asymmetrically and produce another NB and a ganglion mother cell (GMC), which divides symmetrically into two neurons and/or glia cells. Type II NBs also divide asymmetrically but produce another NB and an intermediate neural progenitor (INP), which continues to divide asymmetrically producing INPs and GMCs [4]–[6]. While NBs in the central brain are formed during the embryonic stage, NBs in the so-called optic lobes show a different mode of neurogenssis. The optic lobes are located at the lateral side of each brain lobe and NBs in this region produce neurons for visual processing in the adult stage [7]. There are two proliferating centers in the optic lobe, the outer and inner proliferation center. In both areas, the number of NBs increases during larval stages. Neuroepithelial cells (NE cells) in the outer proliferation center produce lamina and medulla neurons, while NE cells in the inner proliferation center mainly give rise to lobula and lobula plate neurons. In early larval stages, NE cells proliferate by repetitive symmetric cell divisions. In late larval stages, the formation of medulla NBs starts on the medial side of the neuroectoderm and a wave of differentiation progresses from the medial to the lateral side (Figures 1A–1D) [8], [9]. Medulla NBs divide asymmetrically and display a lineage similar to the type I NBs in the central brain.

The basic molecular machanisms of asymmetric cell division are common to all NBs. The Par complex proteins Par3/Bazooka (Baz)-Par6-atypical protein kinase C (aPKC) localize to the apical cortex during mitosis and direct the orientation of the mitotic spindle along the apicobasal axis [10], [11]. During mitosis, Baz-Par6-aPKC regulate the asymmetric localization of three cell fate determinants to the basal cortex. These determintants are inherited only by the GMC, where they stop self-renewal, terminate cell cycle progression, and direct the cell towards differentiation. The set of basaly segregating fate determinants includes the Notch repressor Numb, the NHL-domain protein Brain tumor (Brat), and the homeodomein transcription factor Prospero (Pros) [12]–[16]. In *numb*, *brat*, or *pros* mutants, impaired cell fate determination in larval NBs leads to overproliferation of NBs and transplantation of these mutant brains to the abdomen of adult host flies causes malignant tumors that eventually become metastatic and kill the host [14]–[20].

In a genome-wide RNAi screen for genes regulating proliferation and differentiation in NBs, we identified Daughterless (Da) as a factor controlling NB self-renewal [21]. Da is a class I basic helix-loop-helix (bHLH) protein which forms either a homodimer or a heterodimer with other bHLH proteins and binds to E-box sequences (CANNTG) to regulate transcription of target genes [22]–[25]. During embryonic neurogenesis, heterodimers of Da and Achaete-Scute complex (AS-C) proneural proteins are essential for neuronal precursor formation [26]. *AS-C* is composed of four bHLH transcription factors, namely, Achaete (Ac), Scute (Sc), Lethal of Scute (L(1)sc), and Asense (Ase) [27]–[30]. Since Da expression is ubiquitus, restriced expression of *AS-C* regulates the formation of neural progenitor cells spatially and temporally [31].

In this study, we characterize the role of Da as a tumor suppressor in the *Drosophila* larval brain. We show that inhibiting Da function results in overproliferation of medulla optic lobe NBs and leads to the formation of transplantable brain tumors. We explain this phenotype by showing that Da and Ase promote differentiation through regulating Pros expression, suggesting that the differentiation program is set up in neural stem cells and asymmetric segregation of Pros ensures that the differentiation program is implemented only in one of the two daughter cells. Our data indicate that a regulatory loop between Da/Ase and Pros maintains the balance between self-renewal and differentiation in optic lobe NBs.

## Materials and Methods

### Fly Genetics

Flies were grown at 25°C unless otherwise noted. *w* flies were used as wild-type controls. *da^3^ FRT40A* [32], *ase^1^ FRT19A* [33], *sc^19^ FRT19A* [34], *Df(1)260-1 FRT19A* [35], *FRT82B pros^17^* [12], *UAS-dicer2; insc-Gal4 UAS-mCD8::GFP*, *wor-Gal4 ase-Gal80; UAS-mCD8::GFP*, *dpnOL-Gal4* (Bloomington Stock Center, #47456), *da* RNAi (Vienna Drosophila RNAi Center (VDRC), #51297), *UAS-pros* (a gift from F. Matsuzaki), *hsflp; tub-Gal80 FRT40A; tub-Gal4 UAS-mCD8::GFP*, *ubi-GFP FRT19A; NP7340-Gal4 UAS-flp* [36], *hsflp; act-Gal4 UAS-GFP; FRT82B tub-Gal80* flies were used. For the *da* RNAi or the overexpression of *pros* experiments, F1 pronegy were raised for 1 day at 25°C and shifted up to 29°C. Conditions for transplantation experiments are descried below.

### Histology

Third instar wandaring larvae were dissected in PBS and fixed in 3.7% Formaldehyde in PBS. Samples were washed three times after fixation with PBS containing 0.3% Triton X-100 and transferred to blocking solution (PBS containing 5% normal goat serum and 0.3% Triton X-100). Specimens were incubated with primary antibodies diluted in blocking solution for overnight at 4°C. Primary antibodies were washed four times with PBS containing 0.3% Triton X-100 before the incubation with secondary antibodies for overnight at 4°C. Secondary antibodies were washed four times with PBS containing 0.3% Triton X-100. Specimens were mounted with Vectashield mounting media (Vector) and viewed on a Zeiss LSM710 confocal microscope. Imaris software (Bitplane) was used for preparing three-dimensional images. The following antibodies were provided by Developmental Studies Hybridoma Bank (DSHB): rat anti-Elav (7E8A10, 1∶50), mouse anti-Pros (MR1A, 1∶10). We also used guinea pig anti-Dpn (1∶1000), guinea pig anti-Ase (1∶100), guinea pig anti-Mira (1∶100), rat anti-Ase (1∶50), mouse anti-Da (a gift from C. Cronmiller, 1∶10), rabbit anti-Phospho Histone H3 (Millipore, 1∶1000). Secondary antibodies (Invitrogen) were used at the following dilutions: Alexa Fluor 488 goat anti-guinea pig IgG, 1∶200; Alexa Fluor 488 goat anti-rat IgG, 1∶200; Alexa Fluor 568 goat anti-guinea pig IgG, 1∶200; Alexa Fluor 647 goat anti-rat IgG, 1∶200; Alexa Fluor 647 goat anti-mouse IgG, 1∶200; Alexa Fluor 647 goat anti-rabbit IgG, 1∶200.

### Transplantation of larval brains

Glass needles used in the transplantation experiments were constructed, and a simple micro-injection system was prepared as described previously [19]. 4–6 days old adult *w* females, kept at 25°C, were used as hosts. The host flies were immobilized on an ice-cold metal plate and stuck on a piece of double-sided sticky tape, with their ventral sides up. Crosses were set up at 29°C between virgin females of *UAS-dicer2; insc-Gal4 UAS-mCD8::GFP/CyO* and males of *da* RNAi or *w*. GFP-positive wandering third instar larvae were collected and larval brains were dissected in ice-cold PBS. The dissected brain lobes were transferred into a small drop of cold PBS on a glass microscope slide and cut into two pieces to separate the optic lobes from the central brain. The isolated optic lobes were transplanted into the abdomen of host flies under a GFP microscope to ensure cells were collected by the needle and transplanted into the hosts. After transplantation, host flies were allowed to recover at room temperature for 1–2 hours in fresh standard *Drosophila* medium before transferred to and maintained at 29°C.

## Results

### Da acts as a tumor suppressor in optic lobe neuroblasts

To further characterize the overproliferation caused by *da* RNAi, we induced *da* RNAi by *insc-Gal4* in all larval NBs. The number of Deadpan (Dpn) expressing NBs increased at the expense of Embryonic lethal abnormal vision (Elav) expressing neurons (100%, n = 14) (Figure S1). Although Da was expressed in all NBs of the central brain and in some progenitor cells (Figures S2A–S2B’’’) we did not find any phenotype in these lineages when we induced *da^3^* amorphic mutant clones using mosaic analysis with a repressible cell marker (MARCM) technique [37] (0%, n = 19 for type I NB lineages, and 0%, n = 16 for type II NB lineages) (Figures S2C–S2F’’).

The visual processing centers of the fly brain arise from the so-called optic lobes. The medial surface of the optic lobes is surrounded by medulla NBs that differentiate from NE cells and generate medulla neurons on the inner side of the brain (Figures 1A–1D) [8], [9], [36]. In the optic lobe, Da was expressed in NE cells and in medulla NBs (Figures 1E–1F’’). To induce *da* RNAi in the optic lobe, we used a *dpn*-*Gal4* driver line that showed strong Gal4 expression in NE cells and medulla NBs and weak expression in medulla neurons (Figure S3) (called *dpnOL-Gal4* below, Janelia Gal4 stocks, Bloomington Stock Center #47456) [38]. Expression of *da* RNAi from *dpnOL-Gal4* caused a strong increase of Dpn positive NBs (100%, n = 12) (compare Figures 1G–1G’’ and 1I–1I’’). We also checked the *da* RNAi phenotype with the mitotic marker Phospho-Histone H3 (PH3), the NB marker Miranda (Mira) and the neuronal marker Elav. In the wild type, PH3 positive mitotic cells (NBs and GMCs) were restricted to the periphery of the optic lobe (Figures 1H–1H’’). In *da* RNAi samples, PH3 positive cells were mislocalized and ectopically found in the inner side of the brain (100%, n = 18) (Figures 1J–1J’’). To confirm this phenotype, *da^3^* mutant clones were induced in the optic lobe. In *da^3^* clones, Dpn positive NBs were found in the region that was normally occupied by medulla neurons (92%, n = 26) (Figures 1K–1L). Thus, *da* is required for cell fate determination in medulla NBs.

To test whether the ectopic NBs in *da* RNAi brains have unlimited growth potential and can induce malignant tumors, optic lobes expressing GFP under the control of *insc-Gal4*, were dissected and implanted into the abdomen of wild type adult host flies [19]. Transplanted cells from *da* RNAi brains proliferated and GFP positive cells were observed in the host flies (17%, n = 47), while no substantial growth was observed in control samples (0%, n = 30) (Figures 1M and 1N). PH3 positive mitotically active cells were observed in the tissue from transplanted *da* RNAi samples, and this tumor tissue consisted of both Dpn-expressing NB-like cells and Elav-expressing neuron-like cells (Figure 1O). This suggests that the *da* tumor cells proliferate and some of the cells keep the stem cell state, but these cells also produce differentiating cells. This is consistent with the result from *da^3^* clones, in which both ectopic NBs and differentiated neurons were observed (Figures 1K–1L). From these results, we conclude that Da acts as a tumor suppressor in optic lobe NB lineages.

### Ase regulates NB differentiation during medulla NB development

**Figure 2:**
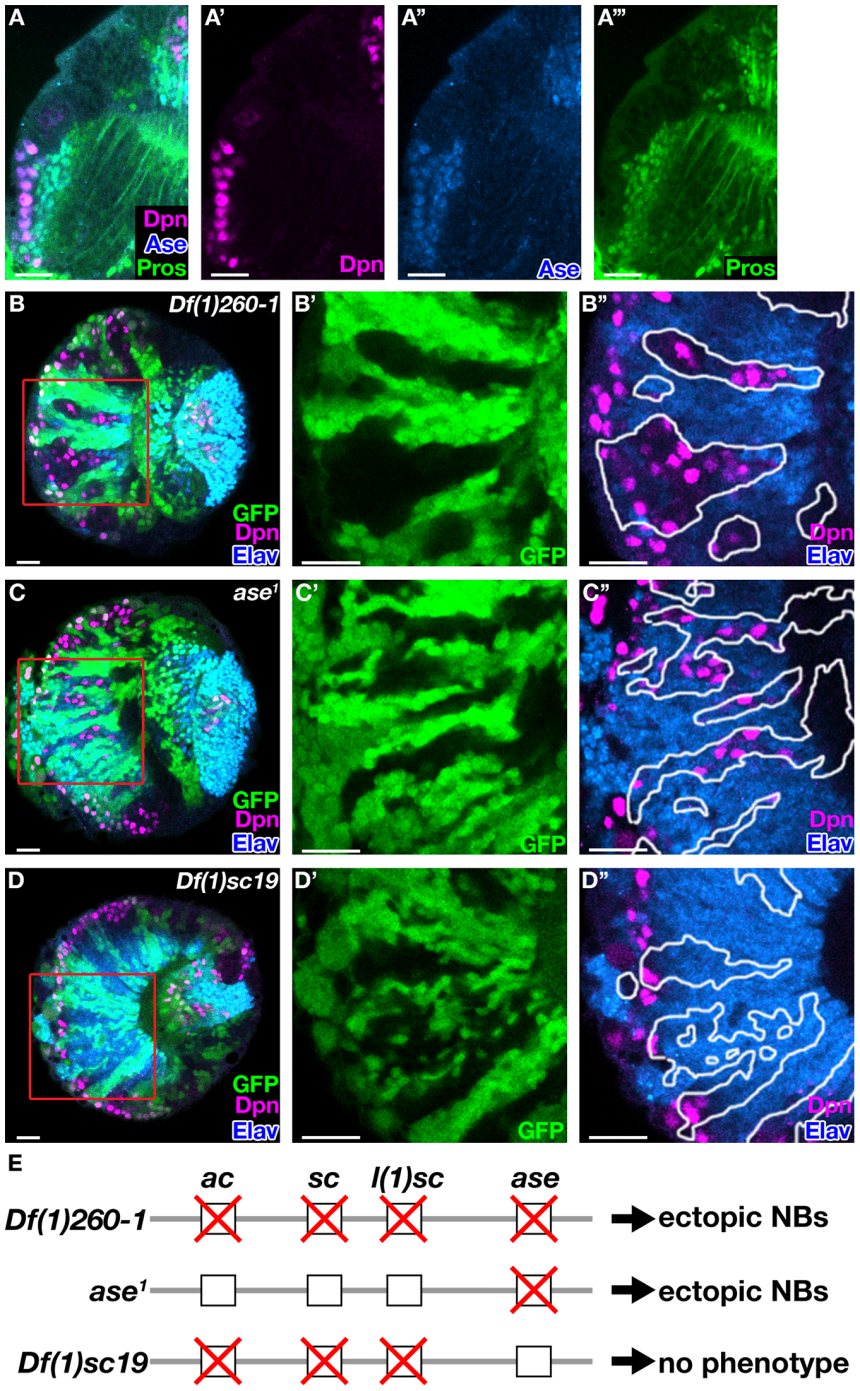
*ase* is required for the cell fate determination in the optic lobe. (A) Expression pattern of Ase and Pros. (B–D’’) Clonal analysis of *Df(1)260-1* (B), *ase^1^* (C), or *Df(1)sc19* (D). (B’, B’’, C’, C’’, D’, and D’’) Enlarged view of the boxed region in (B, C, and D), respectively. Clones are marked by the absence of GFP (B, B’, C, C’, D, and D’) or outlined (B’’, C’’, and D’’). (E) Deficiency lines that uncover *AS-C* region and phenotype summary. Deleted genes are depicted by crosses. Scale bars, 20 μm.

Da is an E-box protein that heterodimerizes with other bHLH type transcription factors, such as the proneural proteins of the AS-C [23], [24], [39]. The AS-C is composed of four transcription factors called Achaete (Ac), Scute (Sc), Lethal of Scute (L(1)sc), and Asense (Ase) [40]. While Ac is not expressed in the optic lobe, three of four AS-C proteins show specific expression [8],[9]. Sc is expressed in the NE cells and NBs, L(1)sc is transiently expressed in the transition zone between NE cells and NBs, and Ase is expressed in NBs and GMCs in the developing medulla (Figures 2A–2A’’’ show expression of Ase) [8], [9]. To test which of the *AS-C* genes might act with Da during cell fate determination in medulla NBs, we induced clones of several deletion lines that uncover the *AS-C* region (Figures 2B–2E, clones in the optic lobe were induced by *NP7340-Gal4* and *UAS-flp* [36]). Ectopic NBs were observed in clones of *Df(1)260-1* uncovering all *AS-C* genes or in *ase^1^* that uncovers the *ase* coding region (84%, n = 57 for *Df(1)260-1* clones, and 88%, n = 73 for *ase^1^* clones) (Figures 2B–2C’’, 2E). On the other hand, no phenotype was observed in clones of *Df(1)sc19*, which deletes *ac*, *sc*, and *l(1)sc* (0%, n = 24) (Figures 2D–2D’’, 2E). Since the phenotype of *Df(1)260-1* or *ase^1^* clones was similar to the phenotype of *da^3^* mutant clones and heterodimerization between Ase and Da has been shown, we conclude that Da acts together with Ase to regulate cell fates in the optic lobe [39]. It has been reported that Da is required for the timly differentiation from NE cells to NBs and L(1)sc is involved in this transition during the optic lobe development [8]. From the expression pattern of *AS-C* genes and results from the clonal analysis using deficiency lines, we propose a dual function for Da: As a heterodimer with L(1)sc, Da promotes the transition from NE cells to NBs. Later, Da acts with Ase in NBs to promote differentiation and prevent tumor formation.

### Pros is a downstream target of Da and Ase

**Figure 3:**
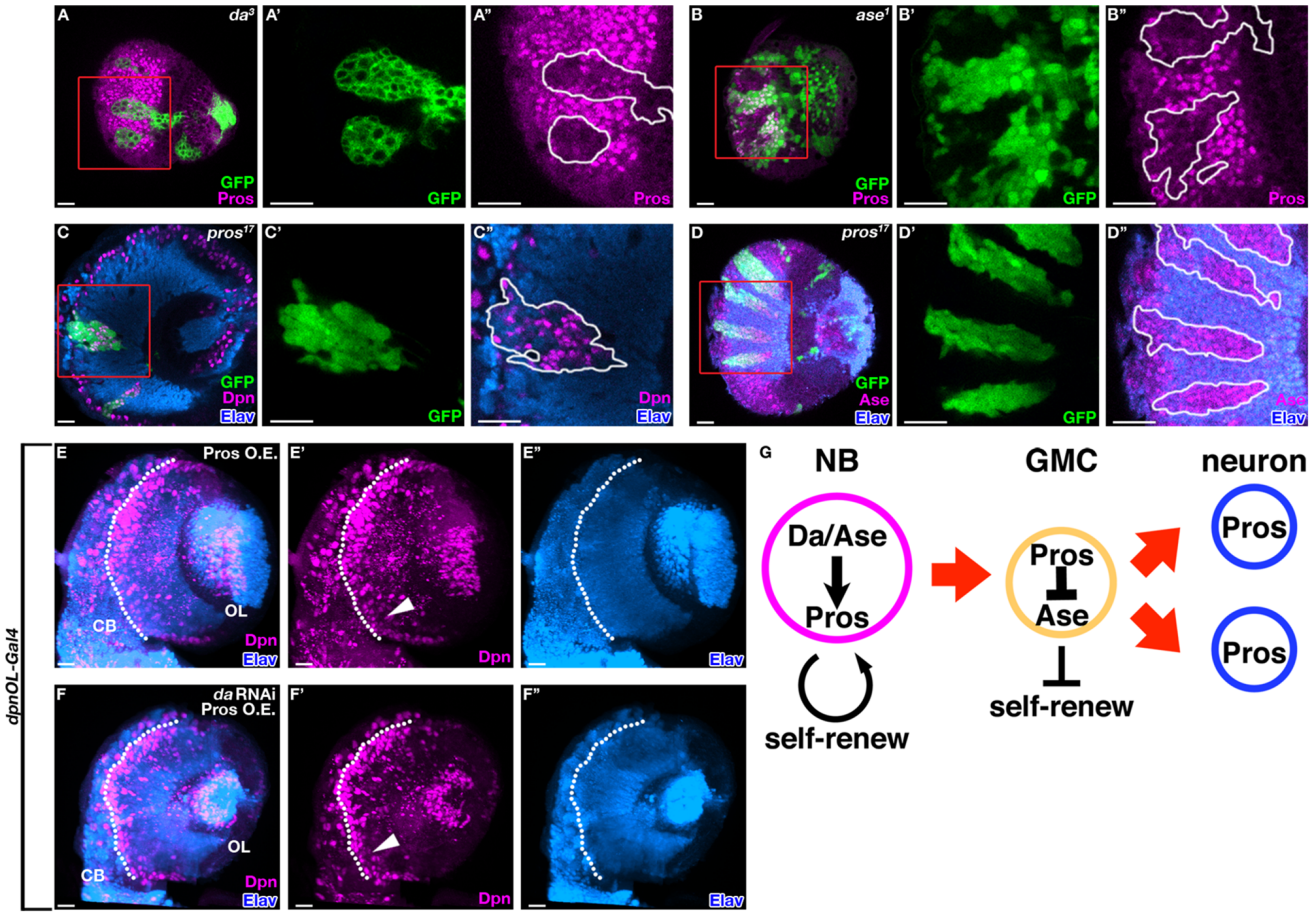
Pros is a downstream target of Da and Ase. (A) Pros expression in *da^3^* clones. (A’ and A’’) Enlarged view of the boxed region in (A). Clones are marked by GFP (A and A’) or outlined (A’’). (B) Pros expression in *ase^1^* clones. (B’ and B’’) Enlarged view of the boxed region in (B). Clones are marked by the absence of GFP (B and B’) or outlined (B’’). (C and D) Ectopic expression of Dpn (C) or Ase (D) in *pros^17^* clones. (C’, C’’, D’ and D’’) Enlarged view of the boxed region in (C) and (D), respectively. Clones are marked by GFP (C, C’, D and D’) or outlined (C’’ and D’’). (E and F) Overexpression of *pros* in WT background (E) or *da* RNAi background (F). *dpnOL-Gal4* was used as a *Gal4* driver. Arrowheads indicate loss of medulla NBs. Dotted lines in (E–F’’) represent the border between the central brain (CB) and the optic lobe (OL). (G) A model for the cell fate regulation by Da, Ase, and Pros. Da and Ase induce Pros expression in NBs. Pros is segregated one of the daughter cells in NB cell division and enters nucleus in GMCs. Pros terminates Ase expression and directs the cell to start differentiation. Scale bars, 20 μm.

To identify the downstream targets of Da and Ase, we tested the expression of candidate genes. The homeodomain transcription factor Pros acts as a cell fate deteminant in embryonic and larval NBs and is regulated by Da and Ase in embryos [12],[15],[16],[26],[41]. In the larval optic lobe, Pros is localized to the basal cortex of dividing NBs and nuclear in GMCs and newly born medulla neurons (Figures 2A–2A’’’) [9], [42]. We tested whether Pros expression is dependent on Da and/or Ase. Pros expression decreased in *da^3^* or *ase^1^* mutant clones (87%, n = 38 for *da^3^* clones, and 74%, n = 38 for *ase^1^* clones) (Figures 3A–3B’’) suggesting that Pros acts downstream of Da and Ase. To test whether *pros* is required for cell fate determination in the optic lobe, we induced *pros^17^* mutant clones. In *pros^17^* mutant clones, ectopic NBs were observed in the medulla neuron layer, which was similar to the phenotype of *da^3^* or *ase^1^* mutant clones (88%, n = 26) (Figures 3C–3C’’). Overexpression of Pros, on the other hand, resulted in a decrease of medulla NBs (100%, n = 8) (Figures 3E–3E’’, compare to Figures 1G–1G’’). To test whether Pros acts downstream of Da, we overexpressed Pros in a *da* RNAi background. A reduced number of medulla NBs were observed in optic lobes overexpressing Pros in a *da* RNAi background, indicating that *pros* is epistatic to *da* (100%, n = 10) (Figures 3F–3F’’). Thus, Pros is a key downstream target of Da and Ase in optic lobe NBs.

Next, we asked whether Pros expression is regulated by Da in the central brain where *da* is not required for NB self-renewal (Figures S2C–S2F’’). Nuclear Pros expression was found in differentiating daughter cells in the wild type. Pros expression remained in *da^3^* mutant clones (0%, n = 11) (Figure S4). Thus, unlike in the optic lobe, Da is not essential for Pros expression in the central brain. This explains why the *da* phenotype is sepecific to the optic lobe NBs, while *pros* mutations cause overproliferation in all larval NBs (Figures 3C–3C’’) [14]–[16]. We speculate that other factors may act redundantly to regulate Pros expression in the central brain.

If Pros is induced by Da and Ase, then how are their functions turned off after asymmetric division? To test whether Pros can terminate the expression of *ase*, we examined Ase expression in *pros^17^* clones. While Ase expression was restricted to the periphery of the optic lobe in wild type, Ase expression continued on the inner side of the optic lobe in *pros^17^* clones (77%, n = 48) (Figures 3D–3D’’). Thus, Pros turns off Ase expression and this transcriptional negative feedback loop regulates the proliferation and differentiation of NBs.

## Discussion

A prevailing view in stem cell biology is that a self-renewal program allows prolonged proliferation in stem cells and is turned off upon differentiation. Our data challenge this view and demonstrate that the ability to differentiate is pre-programmed in neural stem cells. This explains why transcription factors like Da and Ase that are thought to be required for NB specification can be required for proper differentiation and act as tumor suppressors. We propose that a regulatory transcriptional loop assures cell fate determination and inhibits tumor formation (Figure 3G). In a medulla NB, Da and Ase heterodimers induce Pros expression [39] but Pros is excluded from the nucleus and therefore can not terminate Ase expression. After asymmetric cell division, however, Pros enters the nucleus of the GMC where it initiates differentiation and cell cycle exit [43]. In the GMC, Pros terminates Ase expression and therefore triggers an irreversible decision towards differentiation. The data from embryonic NBs suggest that Pros can directly bind to the *ase* region and regulates its expression [43]. In the absence of this regulation, GMCs maintain the stem cell fate and continue to grow into malignant tumors.

The role of Da, Ase, and Pros in neural stem cells could be conserved in mammals. Mammalian class I bHLH genes, namely *E2A* (encoding the E12 and E47 proteins), *E2-2*, and *HEB* are expressed in the developing brain. *E2A*, *HEB*, or *E2A/HEB* transheterozygous mutant mice show a brain size defect, suggesting that class I factors also regulate mouse brain development [44], [45]. Mash1 and Prox1, the vertebrate orthologs of Ase and Pros, are expressed in proliferating neural precursor cells of the developing forebrain and spinal cord [46]. Like in *Drosophila*, Mash1 induces Prox1 and Mash1 promotes an early step of differentiation in neural stem cells [46]. Like in vertebrates, NE cells in the *Drosophila* optic lobe first proliferate by symmetric cell division and then become asymmetrically dividing NBs [8], [9], [47]. From these molecular and developmental similarities, we speculate that the transcriptional regulatory mechanism we have identified might be well conserved in mammalian brains.

Our data are of particular relevance in light of the recently postulated role of stem cells in the formation of malignant tumors [48],[49]. Failure to limit self-renewal capacity in stem cells or defects in progenitor cell differentiation can both lead to the formation of cells that continue to proliferate and ultimately form tumors [50]. While genes acting in stem cells are thought to promote self-renewal, genes required in differentiating cells are thought to promote differentiation and limit proliferation and are therefore candidate tumor suppressors. Our data challenge this view and show that the path to differentiation is initiated in the stem cell and therefore even genes specific to stem cells can act as tumor suppressors. It will be interesting to determine whether a similar mechanism acts in mammalian neural stem cells as well. If it does, the expression pattern of a gene can no longer be used as a main criterium for whether it promotes or inhibits self-renewal in stem cell lineages.

## Supporting Information

Figure S1 ***da*** **RNAi resulted in the overproliferation of NBs.** (A–D) Third instar larval brains for control (A and B) and *da* RNAi (C and D). (B and D) Projection of confocal planes including (A) and (C), respectively. Only Dpn staining is shown. *UAS-dicer2; insc-Gal4*, *UAS-mCD8::GFP* flies were crossed to *w* or *da* RNAi flies. Arrowheads indicate the increase of Dpn expressing cells at the expense of Elav expressing cells. Scale bars, 20 μm.
(TIF)

Figure S2 **Loss of Da function did not alter cell fate in central brain NB lineages.** (A and B) Immunostaining of Da. Anterior view (A) and Posterior view (B) are shown. *insc-Gal4, UAS-mCD8::GFP* marks all NB lineages, while *wor-Gal4, ase-Gal80; UAS-mCD8::GFP* labels type II NB lineage cells. (C, D) MARCM clones in type I NB lineages for control (C) and *da^3^* (D) samples. (E, F) MARCM clones in type II NB lineages for control (E) and *da^3^* (F) samples. (C’, C’’, D’, D’’, E’, E’’, F’, and F’’) Enlarged view of the boxed region in (C), (D), (E), and (F), respectively. Clones are marked by GFP (C, C’, D, D’, E, E’, F, and F’) or outlined (C’’, D’’, E’’, and F’’). Arrows indicate NBs and arrowheads Dpn-positive mature INPs. Dotted lines in (A–B’’, C, and D) represent the border between the central brain (CB) and the optic lobe (OL). The difference of the brain size in (C, D, E, and F) is due to different focal planes where clones are located. Scale bars, 20 μm.
(TIF)

Figure S3 **Expression pattern of the** ***dpnOL-Gal4*** **line.** Anterior view (A), posterior view (B), lateral view (C), and Horizontal view (D) are shown. Expression of *Ga4* was visualized by GFP. Dotted lines in (A–B’’) represent the border between the central brain (CB) and the optic lobe (OL). Arrowheads in (B’ and B’’) indicate Gal4 expression in the central brain. Scale bars, 20 μm.
(TIF)

Figure S4 **Loss of Da function did not change Pros expression in central brain NBs.** (A, B) MARCM clones in type I NB lineages for control (A) and *da^3^* (B) samples. Clones are marked by GFP (A, A’, B, and B’) or outlined (A’’ and B’’). Arrows indicate NBs. Scale bars, 20 μm.
(TIF)
