# Supplementary figures and images for: A Regulatory Transcriptional Loop Controls Proliferation and Differentiation in Drosophila Neural Stem Cells
Source: PLoS One. 2014 May 7;9(5):e97034. doi: 10.1371/journal.pone.0097034 (PMC4013126; doi:10.1371/journal.pone.0097034)

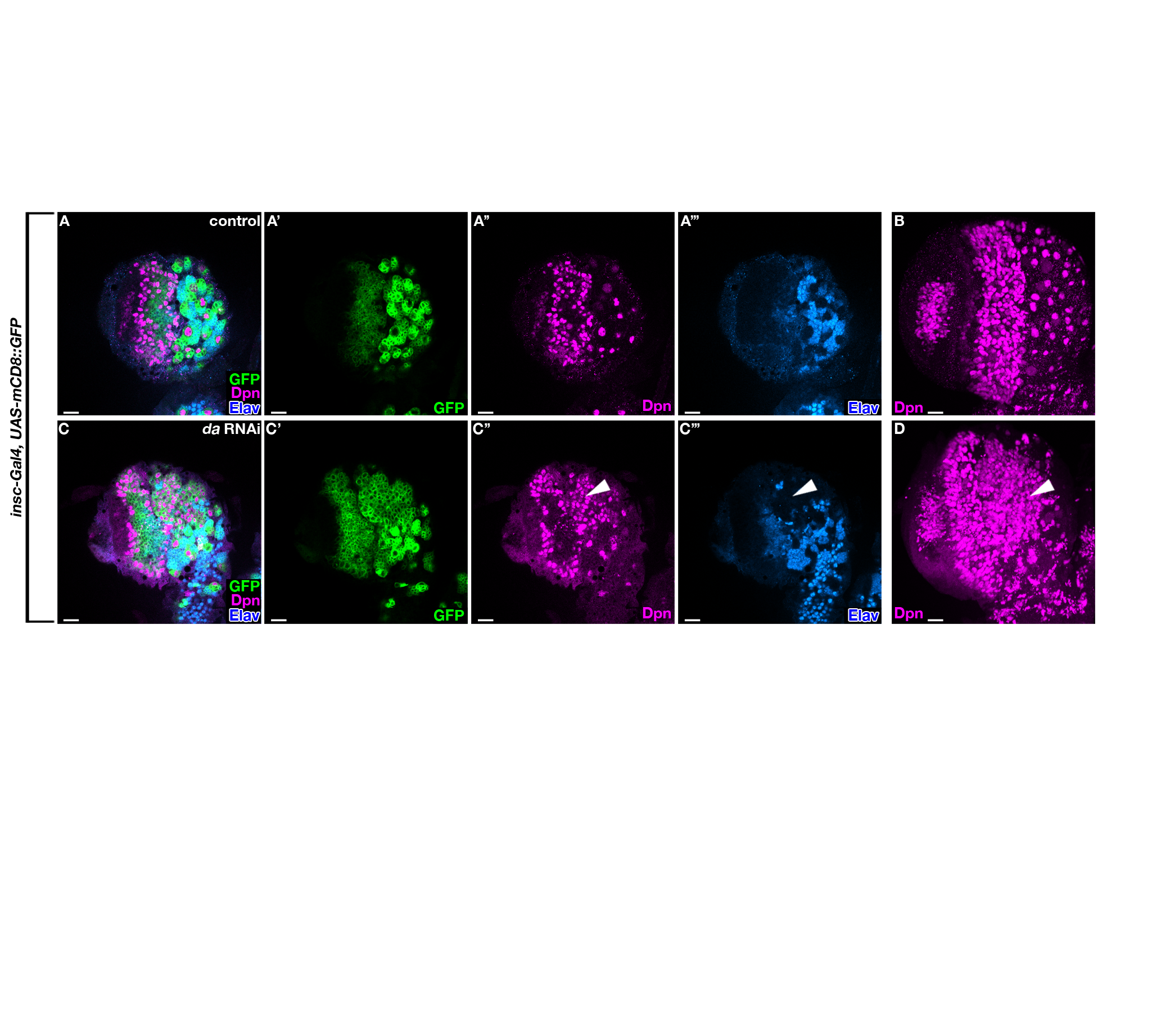

Supplement: Figure S1 — da RNAi resulted in the overproliferation of NBs. (A–D) Third instar larval brains for control (A and B) and da RNAi (C and D). (B and D) Projection of confocal planes including (A) and (C), respectively. Only Dpn staining is shown. UAS-dicer2; insc-Gal4, UAS-mCD8::GFP flies were crossed to w or da RNAi flies. Arrowheads indicate the increase of Dpn expressing cells at the expense of Elav expressing cells. Scale bars, 20 μm. (TIF) [file pone.0097034.s001.tif]

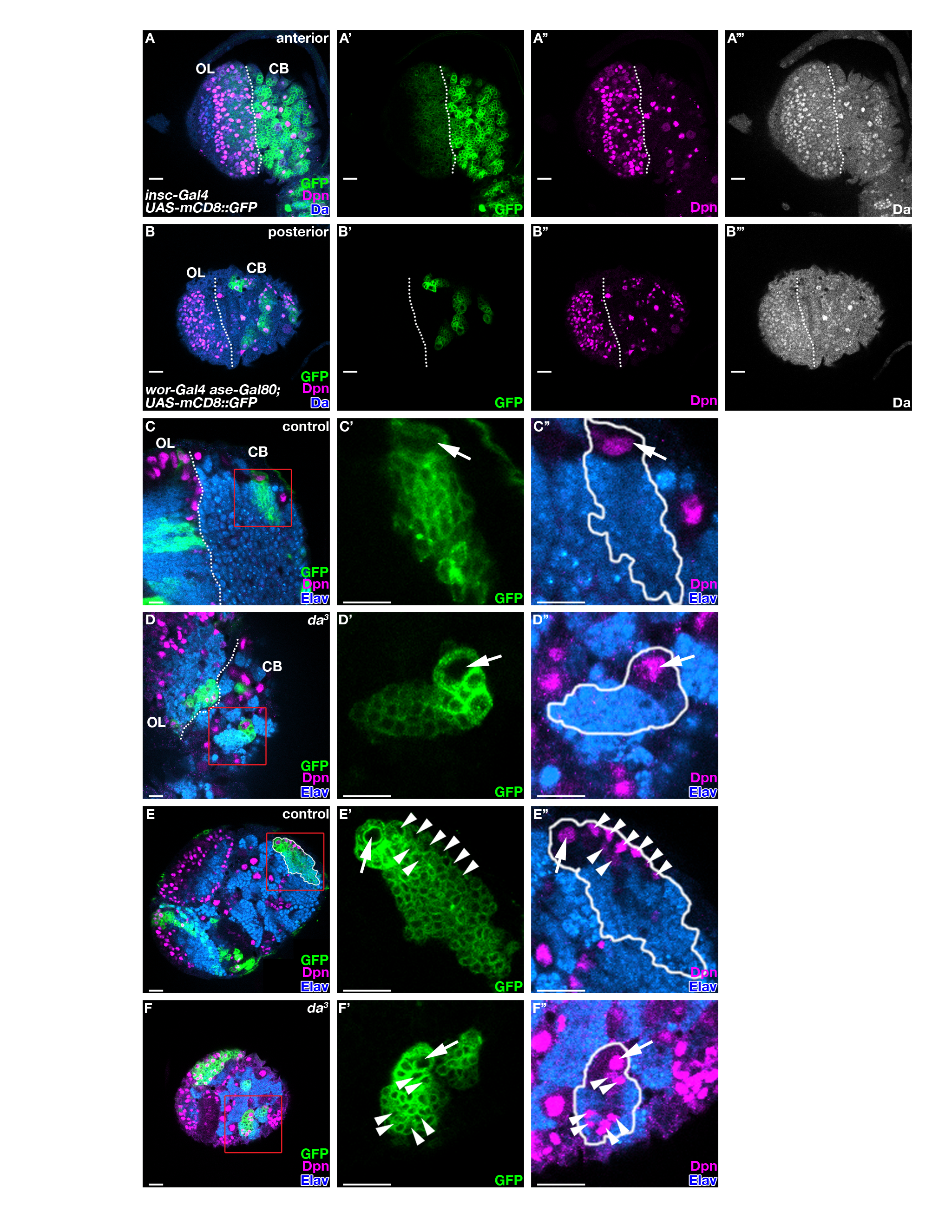

Supplement: Figure S2 — Loss of Da function did not alter cell fate in central brain NB lineages. (A and B) Immunostaining of Da. Anterior view (A) and Posterior view (B) are shown. insc-Gal4, UAS-mCD8::GFP marks all NB lineages, while wor-Gal4, ase-Gal80; UAS-mCD8::GFP labels type II NB lineage cells. (C, D) MARCM clones in type I NB lineages for control (C) and da3 (D) samples. (E, F) MARCM clones in type II NB lineages for control (E) and da3 (F) samples. (C’, C’’, D’, D’’, E’, E’’, F’, and F’’) Enlarged view of the boxed region in (C), (D), (E), and (F), respectively. Clones are marked by GFP (C, C’, D, D’, E, E’, F, and F’) or outlined (C’’, D’’, E’’, and F’’). Arrows indicate NBs and arrowheads Dpn-positive mature INPs. Dotted lines in (A–B’’, C, and D) represent the border between the central brain (CB) and the optic lobe (OL). The difference of the brain size in (C, D, E, and F) is due to different focal planes where clones are located. Scale bars, 20 μm. (TIF) [file pone.0097034.s002.tif]

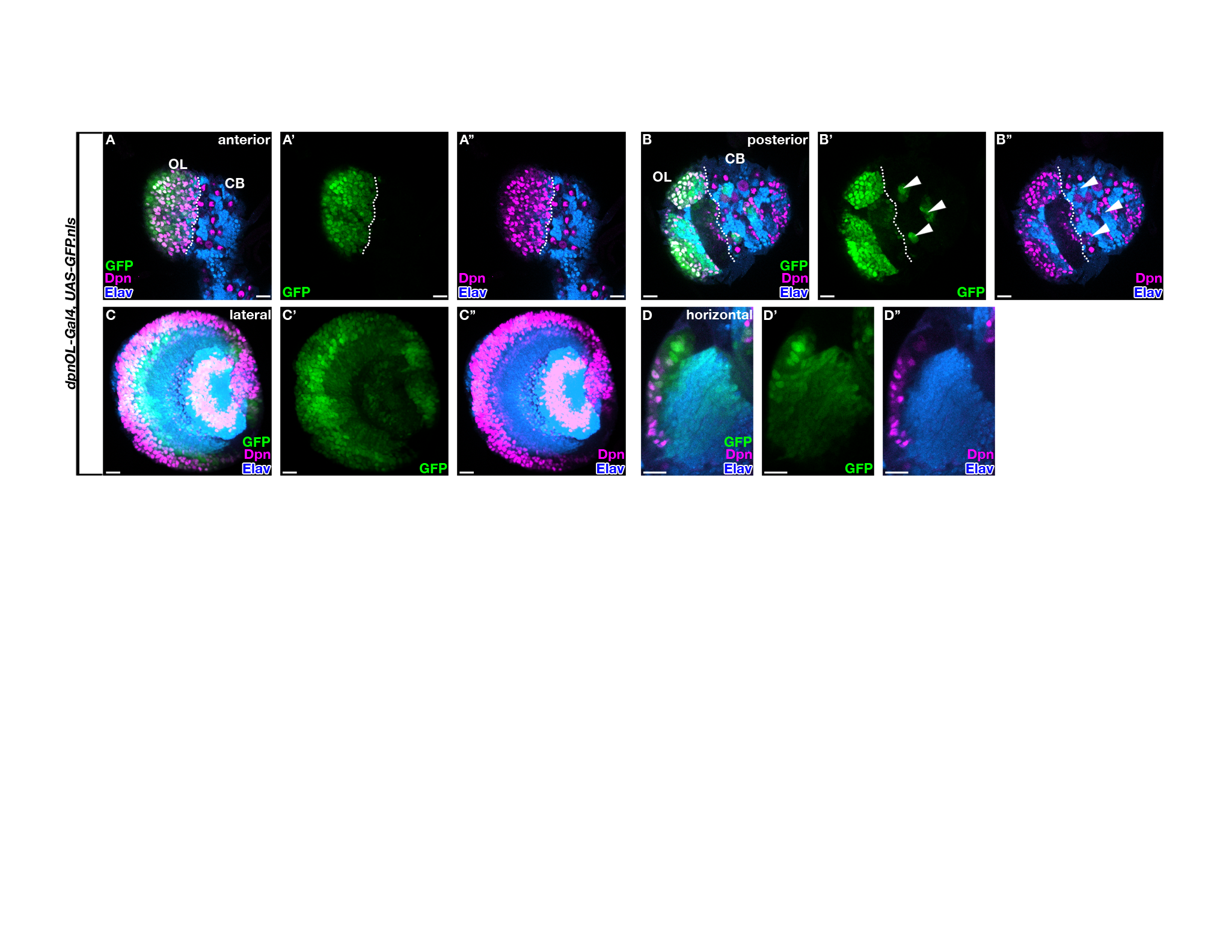

Supplement: Figure S3 — Expression pattern of the dpnOL-Gal4 line. Anterior view (A), posterior view (B), lateral view (C), and Horizontal view (D) are shown. Expression of Ga4 was visualized by GFP. Dotted lines in (A–B’’) represent the border between the central brain (CB) and the optic lobe (OL). Arrowheads in (B’ and B’’) indicate Gal4 expression in the central brain. Scale bars, 20 μm. (TIF) [file pone.0097034.s003.tif]

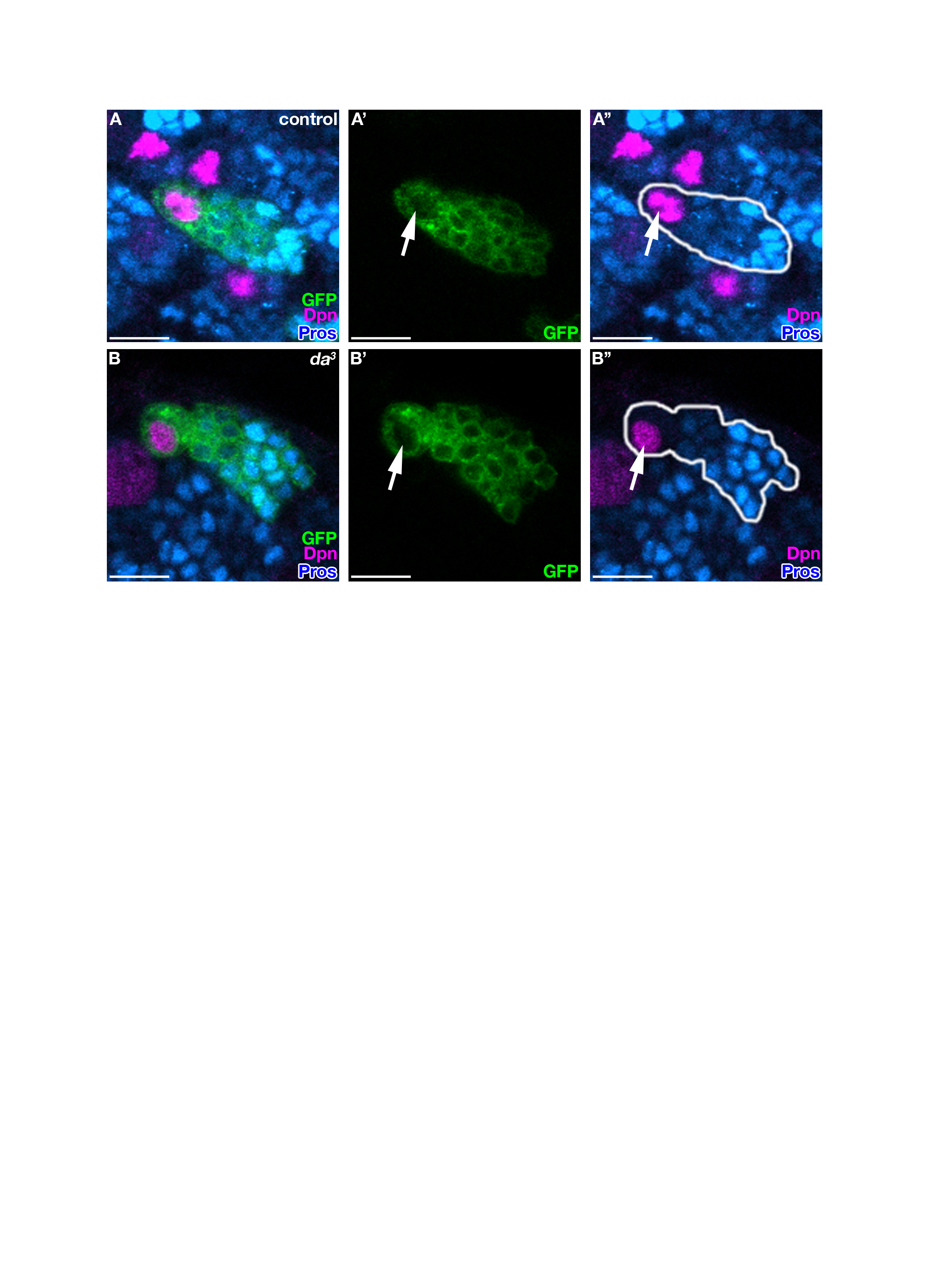

Supplement: Figure S4 — Loss of Da function did not change Pros expression in central brain NBs. (A, B) MARCM clones in type I NB lineages for control (A) and da3 (B) samples. Clones are marked by GFP (A, A’, B, and B’) or outlined (A’’ and B’’). Arrows indicate NBs. Scale bars, 20 μm. (TIF) [file pone.0097034.s004.tif]
